# Prevalence and factors associated with vitamin K prophylaxis utilization among neonates in rural Ethiopia in 2016

**DOI:** 10.1186/s12887-022-03428-6

**Published:** 2022-06-24

**Authors:** Berhan Tsegaye Negash, Yitateku Alelgn

**Affiliations:** Department of Midwifery,Collage of Medicine and Health Science, Hawassa University, Hawassa, Ethiopia

**Keywords:** Vitamin K, Prophylaxis, Neonate, Bleeding, Ethiopia

## Abstract

**Background:**

Neonatal Mortality Ratio (NMR) could not be reversed sufficiently in Ethiopia in the last couple of years. Neonatal bleeding is one of the major causes of neonatal deaths. Administration of vitamin K prophylaxis at birth is the proven strategy to reduce neonatal death which can be caused by vitamin K deficiency bleeding. Although World Health Organization (WHO) recommends universal supplementation of vitamin K prophylaxis for all neonates at birth, many neonates could not get it in many resource poor countries. Despite its importance, information is scarce about uptake of vitamin K prophylaxis in Ethiopia in 2016. Therefore, this study aimed to identify prevalence and factors associated with vitamin K prophylaxis utilization among neonates in Ethiopia in 2016.

**Methods:**

Secondary data analysis of EDHS 2016 was done to assess prevalence and predictors of vitamin K prophylaxis among neonates in Ethiopia five years before EDHS 2016. Multi-stage cluster sampling was used in EDHS 2016. Sample weight and complex analysis were used to minimize bias. Bivariate and multivariable logistic regression analyses were carried out to identify factors associated with vitamin K prophylaxis. Finally, adjusted odds ratio with 95% confidence interval was calculated and *P*-value less than 0.05 taken as the cuff of point for declaration of the statistical significant association.

**Results:**

Prevalence of vitamin K prophylaxis among neonates in Ethiopia in 2016 was found to be 4710(65.5%) in this study. Factors like: Institutional delivery (AOR = 2.2, 95%CI: 1.8, 2.7), neonates from richest family (AOR = 2.1, 95%CI: 1.6, 2.7), neonates from richer household (AOR = 1.4, 95%CI: 1.1, 1.8), starting of antenatal care from 3–6 months of gestational age (AOR = 2.9, 95%CI: 2.3, 3.6) were factors positively associated with vitamin K prophylaxis in Ethiopia.

**Conclusion and recommendation:**

Compared with expected world health organization recommendation of universal supplementation vitamin K prophylaxis, vitamin K utilization is lower among neonates in this study. Hence, it is recommended that strengthen early antenatal care initiation and improving community awareness about vitamin K prophylaxis are the key interventions to improve its uptake. Furthermore, improving institutional delivery might increase uptake of vitamin K prophylaxis.

## Background

Vitamin K is a fat soluble vitamin that can be absorbed from the gastro intestinal tract in the presence of bile salts. Vitamin K is required for the production of coagulation factors II, VII, IX, and X in liver. However, these factors have short half-life, the small stored amounts of vitamin K and inadequate intake of vitamin K can result in deficiency in a short period of time. Inactive precursor proteins, which can be induced in absence of vitamin K, are measurable and can be used as an indicator of vitamin K deficiency [1].Neonates are vulnerable to vitamin K deficiency bleeding since they have low reserves of vitamin K [2, 3].

Vitamin K deficiency bleeding is a coagulopathy which develops later during infancy period if neonates do not get sufficient vitamin K stores to support production of clotting factors. Placental transfer of vitamin K during pregnancy is limited; umbilical cord blood and infant liver reserve levels of vitamin K are substantially below adult levels. As a result, infants are predisposed to develop bleeding disease unless the can get adequate vitamin K prophylaxis during childbirth [4, 5]. Based on the age of its occurrence, Vitamin K deficiency bleeding is classified as: Early, classic, and late [4, 5]. However, for all patterns of bleeding disease, we could prevent it by vitamin K deficiency bleeding through administration of vitamin K prophylaxis at birth [6].

Despite proven effectiveness of vitamin K prophylaxis, there have been concerns about the need for, and safety of vitamin K prophylaxis [4]. However, according to a study report the efficacy and safety of Vitamin K prophylaxis in the prevention of bleeding disease of neonates was approved [7]. The neonatal sources of vitamin K are generally limited to vitamin K prophylaxis given at the time of birth, dietary sources, and intestinal tract bacteria. Specifically, preterm infants should receive large quantities of vitamin K prophylaxis, infant formula and breast milk fortifiers as these neonates cannot easily produce vitamin K or have lowest amount of vitamin K [8]. Vitamin K prophylaxis was observed to reduce bleeding disease of neonates, in the neonatal intensive care unit. Evidence for the administration of prophylaxis vitamin K immediately after birth to prevent bleeding is well established and it is routinely provided to infants in developed countries [5]. On the contrary, once passed at neonatal period, infants do not get vitamin K prophylaxis in Ethiopia.

Although a system of administration of vitamin K prophylaxis in low-resource settings is well established, many health care providers do not know its benefits [9]. Consequently, high neonatal mortality among hospitalized cases has been noted with lack of resources and treatment delay [10]. Based on the previous study report, exclusive breastfeeding, ethnicity, gestational age and women’s age were significantly associated with refusal vitamin K prophylaxis administration [11]. Despite its importance, information is scarce about the burden and predictors of vitamin K prophylaxis administration among neonates in Ethiopia in general. Therefore, this study aimed to assess the prevalence and factors associated with vitamin K prophylaxis among neonates in Ethiopia in 2016.

## Methods

### Study setting

Ethiopia is the third most populous country in Africa with an estimated population of over 100 million. Half of the population are females. Specifically, nearly one fifth (21%) of females are in the reproductive age group (from age 15 to 49 years). Majority of Ethiopian population live in rural areas [12]. Geographically, Ethiopia consists of 9 regions and 2 city administrations. These regions are again divided into 75 zones, 551 districts and 10,000 Kebeles or sub-districts. Ethiopia has endorsed a four-tier health system since 1993. The primary health care unities include: Health posts, health centres and primary hospitals are at primary level, secondary level health facilities included general hospitals, and tertiary services are delivered by specialized hospitals [13].

### Study design, population and period

This study was done based on the analysis of EDHS 2016. EDHS 2016 is national based survey conducted to capture most of the socio-demographic, reproductive and other pertinent health indicators in the diverse population in the community. Women of reproductive age group who delivered a live neonate between within five years before 2016 were included in this study. For woman who gave birth more than once within the study population, the most recent live birth was taken for analysis in this study.

### Sampling technique

Ethiopian demographic health survey is a community based two stage sampling survey conducted every five years. EDHS 2016 is the fourth national survey. The 2007 Ethiopian population census created enumeration areas for counting the population. The lists of these enumeration areas or clusters were utilized as sampling frame for EDHS 2016.

**Fig. 1:**
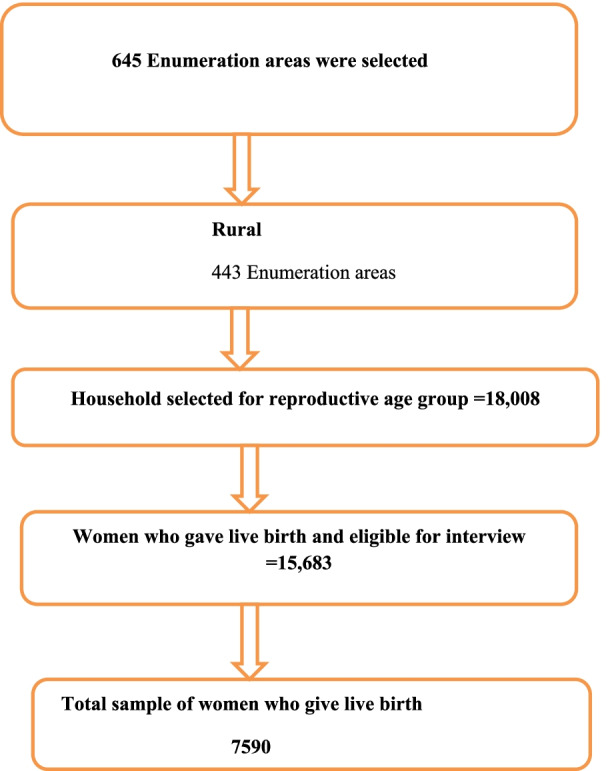
Schematic presentation of sampling strategy of rural women with newborns who give birth in Ethiopia in2016

Figure 1 clearly indicates the schematic presentation of sampling strategy of EDHS 2016. Hence, There were 443 enumeration areas in rural Ethiopia. A fixed number of 28 households per cluster were selected randomly. However, a total of 18,008 households were randomly selected in these rural enumeration areas. There were a total of 15,683 women in the reproductive are groups that were interviewed about different aspects of reproductive health. From these women, we extracted 7590 women who gave live births at least once within five years in 2016 [14]. EDHS 2016 was compiled in different datasets based on the target population of the survey. Individual (women in reproductive age group) data set was used in this study.

### Data collection

EDHS 2016 was conducted from January 18, 2016, to June 27, 2016. This report presents comprehensive, detailed, final outcomes of the survey at the national level, for the nine regional states and two city administrations of Ethiopia. The pre-test was conducted from October 1–28, 2015, in Bishoftu. The DHS Program’s standard Demographic and Health Survey questionnaires were adapted to reflect the population and health issues relevant to Ethiopia. Five questionnaires were used for the 2016 EDHS: Household questionnaire, woman’s questionnaire, man’s questionnaire, biomarker questionnaire, and health facility questionnaire [14]. The woman’s questionnaire was used to extract data from all eligible women. Women who had newborns were selected and analysed.

A four-day field practice was organised, from January 12–15, 2016, to provide trainees with additional hands-on experience before the actual fieldwork. A total of 36 teams were formed for field practice. Each team consisted of a team supervisor, a field editor, three female interviewers, one male interviewer, and two biomarker technicians. Ultimately, 132 individuals were selected as interviewers, 33 as field editors, and 33 as team supervisors.

In the interviewed households, 16,583 eligible women were identified for individual interviews. Interviews were completed with 15,683 women, yielding a response rate of 95%.

### Data analysis

Data were analysed using SPSS version 22 in this study. Before starting of the main analysis, study subjects were extracted or selected from the total of women of reproductive age (individual women file). Important variables were selected, categorized and coded for further analysis. Then, data was weighted to adjust for the problem of either over or under sampling. Weighting variable was created by dividing individual woman variable (V005) by 1,000,000. Subsequently, the univariate analysis of the study subjects was done using the descriptive summary measures. Complex analysis was performed to minimize standard error due to stratification and clustering. This complex analysis includes the following successive steps: First, data weight variable was already created. Second, plan file was created using the following variables: primary sample unit (021), sample strata (v022), and weight created in step 1. Third, sampling with replacement as estimator assumption was fixed. Finally, we have analysed the plan file created in these three steps to identify factors associated with vitamin K prophylaxis intake. Bivariate analysis or first order analysis was also conducted using the Pearson chi-square test statistics to show the presence of association. Data were further analyzed through binary and multivariate logistic regression respectively to identify predictors of vitamin K prophylaxis through complex analysis. *P*-value less than 0.25 and 0.05 were taken as a cut of point to declare the statistical association between the outcome and explanatory variables. Manual backward stepwise method was used as predictor reduction in multivariable logistic regression model. Model fitness was checked using chi-square goodness of fit test and found less than 0.05. Descriptive statistics was conducted by the use of texts, tables and charts.

### Socio-demographic characteristics

Table 1 of this study indicated that from a total of 7190 the women who gave birth in the past five years prior to the survey, all women were participated in this study making a response rate of 100%. The mean age of the respondent is 29.26 ± 6.8 years. Majorities 5530 (72.9%) were in the age range of less than 20 years. More than half, 4791(63.1%), of study subjects attended elementary school. Whereas, only few 230(3.0%) were attended higher education. The proportion of study subjects, who follow orthodox Christianity, 2882 (38%), is almost as equal as Muslim followers. However, only few 97(1.3%) study participants have traditional belief and a few 64(0.8%) believe by other type of religion. Majorities of the study participants 7109(93.7%) were married (See Table 1).

**Table 1:** Socio-demographic characteristics of the study participants (*N* = 7590)

Variable	Un-weighted n (%)	Weighted n (%)
**Age**		
Less than 20	5108(71)	5530(72.9)
Above 20 years	2085(29)	2060(27.1)
**Region**		
Tigray	7729(10.7)	537 (7.1)
Afar	647(9.0)	71 (0.9)
Amhara	764(10.6)	1632 (21.5)
Oromia	1031(14.3)	3129 (41.2)
Somali	806(11.2)	269 (3.5)
Benishangul	576(8.0)	81 (1.1)
SNNPR	893(12.4)	1601(21.1)
Gambela	534(7.4)	21 (0.3)
Harari	411(5.7)	17(0.2)
**Education**		
No education	4359(60.6)	4791(63.1)
Primary	1942(27)	2150(28.3)
Secondary	577(8)	420(5.5)
Higher	315(4.4)	230(3.0)
**Religion**		
Orthodox	2369(32.9)	2882(38.0)
Catholic	49(0.7)	71(0.9)
Protestant	1338(18.6)	1651(21.8)
Muslin	3324(46.2)	2824(37.2)
Traditional	64(0.9)	97(1.3)
Other	49(0.7)	64(0.8)
**Wealth index**		
Poorest	2473(34.4)	1609(21.2)
Poorer	1348(18.7)	1599(21.1)
Middle	1228(17.1)	1602(21.1)
Richer	1092(15.2)	1449(19.1)
Richest	1052(14.6)	1330(17.5)
**Marital status**		
single	147(2.0)	153(2.0)
married	6662(92.6)	7109(93.7)
widowed	106(1.5)	95(1.3)
divorced	278(3.9)	233(3.1)
**Working status**		
not working	4077(56.7)	4078(53.7)
working	3116(43.3)	3512(46.3)
**Place of delivery**	1512(21)	969(12.8)
	5681(79)	6621(87.2)

### Reproductive and health seeking behaviour charactestics

Based on the report of Table 2 of this study, most of the study subjects, 3350 (44.1%), started antenatal care visit above six months. Majorities (72.3%) of women did not listen radio at all. Nearly two women out of five had mobile cell phone. A few 769(10.1%) study participants were pregnant at the time of interview. Only 680(9%) of the study subjects had termination of pregnancy. Most of the study subjects, 5066(66.8%), gave birth at home. A majorities of women 6519(85.9%) had up to six children (See Table 2).

**Table 2:** Reproductive and health seeking behaviour of the study participants (*N* = 7590)

Variable	Un-weighted N (%)	Weighted N (%)
**Time of ANC visit**		
Within three months	1808(25.1)	1545 (20.4)
Three to six months	2460(34.2)	2695 (35.5)
more than six months	2925(40.7)	3350 (44.1)
**Frequency of listening radio**		
Not at all	5343(74.3)	5491(72.3)
Less than once a week	924(12.8)	1030(13.6)
At least once a week	926(12.9)	1069(14.1)
**Frequency of reading newspaper**		
Not at all	6590(91.6)	7050(92.9)
Less than once a week	462(6.4)	404(5.3)
At least once a week	141(2.0)	135(1.8)
**Frequency of watching television**		
Not at all	5511(76.6)	6102(80.4)
Less than once a week	628(8.7)	764(10.1)
At least once a week	1054(14.7)	724(9.5)
**Mobile ownership**		
No	5312(73.8)	6217(81.9)
Yes	1881(26.2)	1373(18.1)
**Current pregnancy**		
No	6414(89.2)	6820(89.9)
Yes	779(10.8)	769(10.1)
**Abortion**		
No	6556(91.1)	6910(91.0)
Yes	637(8.9)	680(9.0)
**Place of delivery**		
home	4395(61.1)	5066(66.8)
HI	2798(38.9)	2523(33.2)
**Number of children**		
Up to six	6252(86.9)	6519(85.9)
More than six	941(13.1)	1071(14.1)

## Prevalence of utilization of vitamin K prophylaxis

The overall prevalence of utilization of vitamin K prophylaxis is 4695(65.3%) among neonates in Ethiopia from from 2011 to 2016. The rest of 2495(34.7%) of neonates did not get vitamin K prophylaxis in Ethiopia.

### Regional difference of uptake of vitamin k prophylaxis

**Fig. 2:**
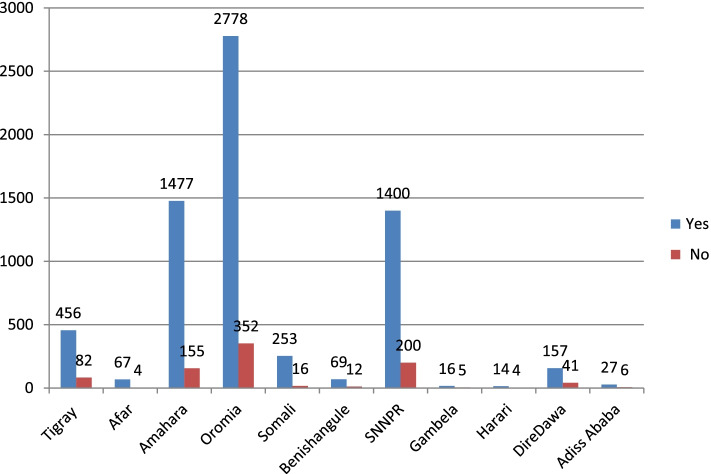
Regional difference of vitamin k supplementation among newborns in Ethiopia in 2016

In Ethiopia there are 9 administration regions. Based on the report of Fig. 2, Harari region accounts the least level of vitamin K prophylaxis 14(0.0001%). Whereas, neonates in Oromia region took highest prophylaxis which account 2778(36.5%) among nine Ethiopian regions.

### Factors associated with vitamin K prophylaxis

Based on the report of Table 3, seven factors showed statistically significant association in this study: Region, wealth index, antenatal care, mobile ownership, frequency of listening radio and awareness of danger sign. On the contrary, only three variables showed statistically significant association in multivariate logistic regression. Place of delivery, wealth index, antenatal care visits are factors which affect utilization of vitamin K prophylaxis among newborns. Neonates of urban women were 2.2 times more likely to take vitamin K utilization than their counterparts (AOR = 2.2, 95%CI, 1.8, 2.7). The odds of uptake of vitamin K supplementation among newborns of richer and richest women were 2.1 (AOR = 2.1, 95%CI, 1.6, 2.7) and 1.4 times (AOR-1.4, 95%CI, 1.1, 1.8) than newborns of poorest women respectively. The chance of neonates of women who start antenatal care three to six months were 2.9 (AOR, 95%CI, 2.3, 3.6) more than neonates of women who initiate their antenatal care beyond six months of pregnancy (See Table 3).

**Table 3:** Factors associated with Vitamin k administration among new-born in Ethiopia (EDHS-2016)

**Variable**	*p*-value	COR(95%CI)	*P*-Value	AOR(95%CI)
**Place of delivery**				
Home	0.000	**Ref**	0.000	**Ref**
Health institution	0.000	2.8(2.5,3.3)*	0.000	2.2 (1.8,2.7)**
**Wealth index**				
Richest	0.000	2.6(2.2,3.4)*	0.000	
Richer	0.000	1.6(1.3, 2.0)*	0.002	
rich	0.108	1.2(0.9,1.5)	0.346	1.1 (0.9,1.4)
Poorer	0.026	1.3(1.1,1.6)*	0.064	1.2 (0.9,1.5)
Poorest	0.00	**Ref**	0.000	**Ref**
**ANC visit**				
Less than 3 months	0.566	0.9 (0.8,1.13)	0.346	0.9 (0.8,1.1)
Three to six months	0.000	3.6 (2.9,4.4)*	0.000	2.9 (2.3,3.6)**
More than 6 months	0.00	**Ref**	0.000	**Ref**
**Own mobile**				
No	0.00	2.1(1.8,2.5)*	0.55	1.1 (0.9,1.3)
Yes	0.00	**Ref**	0.009	**Ref**
**Freq. listening radio**				
Not at all	0.000	1.7(1.4,2.02)*	0.87	0.97(0.8,1.2)
Less than once/week	0.238	1.2(0.9,1.5)	0.82	0.97(0.8,1.3)
More than once/week	0.000	**Ref**		**Ref**
**Region**				
Tigray	0.00	**Ref**		**Ref**
Afar	0.034	3.1(1.1,9.0)*	1.031	1.04(0.41,2.6)
Amhara	0.000	1.7(1.3,2.3)*	1.922	1.94(0.49,7.73)
Oromia	0.009	1.4(1.1,1.8)*	1.423	1.42(0.57,3.51)
Somali	0.000	2.9 (1.7,5.1)*	0.973	0.97(0.39,2.39)
Benishangul	0.845	1.1 (0.55,2.1)	1.577	1.59(0.57,4.49)
SNNPR	0.114	1.3 (0.95,1.7)	0.810	0.81(0.27,2.43)
Gambela	0.328	0.6 (0.21,1.7)	1.002	1.00(0.40,2.48)
Harari	0.527	0.7 (0.2,2.2)	0.553	0.56(0.14,2.23)
Dire-Dawa	0.080	0.7 (0.5,1.1)	0.822	0.83(0.18,3.68)
Addis Ababa	0.505	0.7 (0.3,1.8)	1.637	1.65(0.63,4.28)
**Awareness of new born danger sign**				
No	0.000	**Ref**	2.10	**Ref**
Yes	0.000	1.71(1.25,2.35)*	0.90	1.02(0.73,1.42)

KEY: *= p-value <0.25, **=p-value <0.05, *Ref* = Reference

## Discussion

This study showed that prevalence and factors associated with vitamin K prophylaxis administration of the neonates in Ethiopia in 2016. Neonates are at risk for vitamin K deficiency bleeding (VKDB) which is caused by inadequate prenatal storage and deficiency of vitamin K in breast milk [15]. Despite recommendation of Ethiopian national obstetrics care guideline for universal prophylaxis vitamin K prophylaxis, many neonates fail to access vitamin K prophylaxis at birth which is done either in health institution or home delivery [16]. Although information about vitamin K prophylaxis is valuable for skilled delivery care providers, researchers and policy makers, no previous study was conducted on prevalence and factors associated with vitamin K prophylaxis in Ethiopia. Hence, this study can provide comprehensive information about prevalence and factors associated with vitamin K prophylaxis in Ethiopia in 2016.

Based on the report of this study, the overall prevalence of vitamin K prophylaxis is found to be 65.5% (95%CI = 64.9%, 66%) among Ethiopian neonates, in2016. This finding is in line with a study done in Uganda (66%) [17]. Compared to WHO recommendation, the prevalence of vitamin K prophylaxis among neonates in Ethiopia is low [18, 19]. This might be associated with the fact that most deliveries are conducted at home in rural Ethiopia in 2016. Furthermore, most health facilities are suffered from resource deficiencies including vitamin k prophylaxis.

According to this study finding, neonates were delivered from richest women were more likely to obtain vitamin K prophylaxis than neonates delivered form poor family members. This may be due to the fact that richest mothers are more likely to deliver in health institution than poorest mothers due to transportation and they are more like to have awareness about institutional delivery and vitamin K prophylaxis. The other may be richest family are able to pay for medication in the absence of vitamin K in public health institutions. This finding supported by study conducted in Alberta which concludes mothers who deliver at home were more likely to refuse vitamin K administration for their neonates [20]

Neonates who were delivered in health institutions are more likely to take vitamin k prophylaxis than their counterparts. This may be due the fact that vitamin K prophylaxis is one of the components of essential newborn care in Ethiopia. Hence, every neonate can access vitamin K prophylaxis immediately after delivery. However, vitamin K prophylaxis cannot be given in all health facilities due to several reasons. For instance, drug might not be available in health institutions of rural areas [21]. It might not be effectively stored due to lack of standardized storage such as refrigerator. Moreover, most of clean deliveries are conducted by health extension workers in rural Ethiopia. Health extension workers are clean delivery attendants who are not eligible to give parenteral medication like vitamin K injection. Neonates delivered from mothers who start antenatal care before three month were more likely to gate vitamin K prophylaxis than neonates delivered from mothers who start antenatal care after six months. Although no previous study to compare the finding, this association might be due to increased chance of institutional delivery in newborn of women who attend antenatal care than their counterparts [19].

### Limitation

The current study had much strength. First, it is the original research which laid down the foundation for other studies. Second, this study was national representative study with good validity and reliability. However, this study had also a number of limitations recalling bias and lack of comparative studies were the potential limitation of this study. Furthermore, this study could not see the provider perspective and health institution factors which can influence vitamin K prophylaxis.

## Conclusion

Prevalence of vitamin K prophylaxis utilization is low among neonates compared to national target in 2016. Neonates of rich, urban women; and women who start antenatal care early have better chance of receiving vitamin K prophylaxis.

### Practical implication

The current study had both theoretical and practical implications. Theoretically, women socio-demographic and reproductive factors, which are associated with uptake of vitamin K prophylaxis, are established. Practically, skilled delivery care providers should use this finding to increase the coverage of vitamin K prophylaxis.

### Recommendation

#### For policy makers

The authors could recommend improving the socio-economic status of women. Furthermore, counselling about cares given during labor like vitamin K and its importance should be integrated into antenatal care.

#### Birth attendants

Birth attendants should strongly advice about new born care components at birth during the prenatal care follow up.

#### Future researcher

The future researcher should focus on factors associated in the health facility and providers.

## Data Availability

Permission to access database was officially obtained. The database was available at an official website of DHS which is at https://dhsprogram.com.

## Abbreviations

CI: Confidence interval
DHS: Demographic and health survey
COR: Crude odds ratio
EA: Enumeration area
ANC: Ante-natal care
CSA: Central statistical agency

## References

[1] Edstrom C, Christensen R, Andrew M (2000). Developmental aspects of blood hemostasis and disorders of coagulation and fibrinolysis in the neonatal period. Hematol Prob Neonate.

[2] Blackmon L, Batton DG, Bell EF, Engle WA (2003). Controversies concerning vitamin K and the newborn. Pediatrics.

[3] Pichler E, Pichler L (2008). The neonatal coagulation system and the vitamin K deficiency bleeding–a mini review. Wien Med Wochenschr.

[4] Zipursky A (1999). Prevention of vitamin K deficiency bleeding in newborns. Br J Haematol.

[5] American Academy of Pediatrics Vitamin K Ad Hoc Task Force (1993). Controversies concerning vitamin K and the newborn. Pediatrics.

[6] Poddar B (1997). Early hemorrhagic disease of newborn. Indian Pediatr.

[7] Bindra A (1986). Role of vitamin K prophylaxis in newborn. Padiatrie und Padologie.

[8] Greer FR (2010). Vitamin K the basics—what's new?. Early Human Dev.

[9] Danielsson N, Hoa DP, Thang NV, Vos T, Loughnan PM (2004). Intracranial haemorrhage due to late onset vitamin K deficiency bleeding in Hanoi province. Vietnam Arch Dis Child Fetal Neonatal Ed.

[10] Shearer MJ (2009). Vitamin K deficiency bleeding (VKDB) in early infancy. Blood Rev.

[11] Loyal J, Taylor JA, Phillipi CA, Goyal NK, Wood KE, Seashore C, King B, Colson E, Shabanova V, Shapiro ED (2018). Factors associated with refusal of intramuscular vitamin K in normal newborns. Pediatrics.

[12] Central Statistical Authority AA (2012). 2007 Population and housing census of ethiopia administrative report.

[13] Ethiopia FMOH. Health Sector Development Program IV 2010/11 – 2014/15. 2010.

[14] Csa I (2016). Central statistical agency (CSA)[Ethiopia] and ICF. Ethiopia demographic and health survey, Addis Ababa, Ethiopia and Calverton, Maryland, USA.

[15] Ng E, Loewy AD (2018). Guidelines for vitamin K prophylaxis in newborns. Paediatr Child Health.

[16] Ethiopia FMOH. Management protocol on selected obstetrics topics. 2010. 20. IIPS, I.C.F. India National Family Health Survey NFHS-4 2015–16. Mumbai: IIPS and ICF; 2017. S.1255-9.

[17] Siedner MJ, Mwanga-Amumpaire J, Shearer MJ, Harrington DJ, Wariyar U (2015). Prevalence and predictors of functional vitamin K insufficiency in mothers and newborns in Uganda. Nutrients.

[18] Victora CG, Van Haecke P (1998). Vitamin K prophylaxis in less developed countries: policy issues and relevance to breastfeeding promotion. Am J Public Health.

[19] AlemiKebede KH, Teklehaymanot AN (2016). Factors associated with institutional delivery service utilization in Ethiopia. Int J Women's Health.

[20] Khambalia AZ R, CL, Bowen JR, Nassar N: Vitamin K Refusal at Birth in Alberta. J Paediatr Child Health 2012;48(8):665–668 2014. 10.1111/j.1440-1754.2012.02448.x 22515745

[21] Iips I (2017). National Family Health Survey (NFHS-4), 2015–16.

